# The Association of Vitamin D Levels and Dental Caries in Older Adults: A Cross-Sectional Study

**DOI:** 10.3390/nu16142307

**Published:** 2024-07-18

**Authors:** Man Hung, Amir Mohajeri, Mahsa Sadri, Elahe Khodabandeh, Ibrahim Zeitoun, Martin S. Lipsky

**Affiliations:** 1 College of Dental Medicine, Roseman University of Health Sciences, South Jordan, UT 84095, USA; amohajeri@roseman.edu (A.M.); izeitoun254@student.roseman.edu (I.Z.);; 2 Division of Public Health, University of Utah, Salt Lake City, UT 84112, USA; 3 Department of Orthopaedics, University of Utah, Salt Lake City, UT 84112, USA; 4 Department of Population Health Sciences, University of Utah, Salt Lake City, UT 84112, USA; 5 School of Business, University of Utah, Salt Lake City, UT 84112, USA; 6 The Wharton School, University of Pennsylvania, Philadelphia, PA 19104, USA; 7 Huntsman Cancer Institute, Salt Lake City, UT 84112, USA; 8 Institute on Aging, Portland State University, Portland, OR 97201, USA

**Keywords:** vitamin D, dental caries, older adults, NHANES, DMFT, public health, oral health

## Abstract

Introduction: Most research examining vitamin D and dental caries focuses on children and younger adults. This study investigated the association between vitamin D levels and dental caries in older adults using data from the United States National Health and Nutrition Examination Survey from 2011 to 2016. Methods: Data were analyzed from 2723 participants aged 65 years and older who completed both dental examinations and serum 25(OH)D tests. Dental caries assessments included the decayed, missing, and filled teeth (DMFT) index and the presence of untreated dental caries. Vitamin D levels were measured as serum 25(OH)D concentrations and categorized as severely deficient (<25 nmol/L), deficient (25–49.9 nmol/L), insufficient (50–74.9 nmol/L), and normal (≥75 nmol/L). Logistic regression and Poisson regression models were used to assess the association between vitamin D levels and dental caries, adjusting for demographic factors. Results: The mean DMFT score was 17.73 ± 8.34, with 35.1% of participants having untreated dental caries. Vitamin D deficiency was associated with a 1.44 times higher likelihood of untreated caries (95% CI: 1.15, 1.81), which weakened after adjustment for demographic factors (adjusted OR: 1.23, 95% CI: 0.97, 1.55). Severe vitamin D deficiency correlated with a 1.13 times higher DMFT score (95% CI: 1.06, 1.20), with the association remaining similar after adjustment (adjusted RR: 1.12, 95% CI: 1.05, 1.20). Significant differences in vitamin D levels were observed across gender, race/ethnicity, and country of birth. Conclusions: This study suggests the potential importance of adequate vitamin D levels for maintaining dental health among older adults. Vitamin D deficiency is associated with a higher risk of poorer DMFT scores. Public health strategies that include vitamin D screening and supplementation, particularly for high-risk groups, may improve oral health outcomes in the older adult population. Further research is needed to elucidate the mechanisms by which vitamin D influences dental health and the potential for vitamin D supplementation to reduce the burden of dental caries in older adults.

## 1. Introduction

Tooth decay, also known as dental caries, is a widespread chronic disease primarily caused by tooth-adherent cariogenic bacteria, notably *Streptococcus mutans*, *Lactobacilli*, and *Streptococcus sobrinus* [1,2]. These bacteria metabolize dietary sugars, producing acids as metabolic byproducts. The resultant acidic environment gradually causes the demineralization of the tooth structure over time [3]. The progression of dental caries is heavily influenced by alterations in the biofilm microbiota within the oral cavity. Frequent sugar consumption shifts the oral environment towards a more acidogenic and aciduric state, favoring the proliferation of acid-producing bacteria [4]. This shift in the biofilm’s microbial composition disrupts the natural balance, promoting conditions that enhance acid production. As the acidic environment persists, it facilitates a net loss of minerals from the tooth’s hard tissues, such as enamel and dentin; over time, this demineralization process can lead to the formation of visible carious lesions, commonly seen as cavities or holes in the teeth [5].

Histologically, carious tissue can be divided into four distinct zones, with three of these zones being visible clinically [1]. The outermost layer is a necrotic and contaminated zone, which contains a microbial biofilm with a high microbial load. This layer is heavily infected and represents the most deteriorated part of the carious lesion. Beneath this is the zone of demineralization. This area is characterized by an anaerobic atmosphere, which fosters further breakdown of the tooth structure. Clinically, this zone correlates with what is referred to as “leathery dentine”, a softer, more pliable form of dentine that can be easily excavated during dental procedures. The innermost zone, which lies near the pulp, is called the translucent zone. This zone comprises firm but softer dentine and is characterized by demineralization. Notably, this area is free from microorganisms due to their inability to penetrate to such depths [6]. This lack of microbial presence is a key differentiator from the more superficial zones of the carious lesion. The prognosis of dental caries is multifactorial and depends on the patient’s overall health, oral hygiene practices, and the extent and severity of the carious lesions [7]. Early intervention plays a crucial role in the management of dental caries. Radiographic interpretation and clinical assessment such as visual and tactile perceptions are considered conventional methods for caries assessment. Using these methods would provide a better estimation of lesion depth [8]. Caries risk assessment (CRA), such as CAMBRA (Caries Management by Risk Assessment), is also an important tool for the successful management of dental caries. This evidence-based approach assesses factors that contribute to caries and can help design treatment plans to arrest dental caries in the early stages and prevent cavity formation [9]. For example, in its initial stages, carious lesions can often be reversed through remineralization strategies, such as fluoride treatments and improved oral hygiene. However, once decay progresses to more advanced stages, restorative treatments become necessary to repair the damaged tooth structure [10]. These treatments may include fillings, crowns, or more extensive procedures like root canals, depending on the severity of the decay.

Vitamin D, a fat-soluble vitamin, is essential for regulating calcium and phosphate metabolism [11], which is essential for maintaining healthy bones and teeth. The primary sources of vitamin D include exposure to sunlight, dietary intake, and supplementation [12]. This vitamin promotes the absorption of calcium and phosphate and contributes to the mineralization and overall health of the skeletal system. Research has established a significant link between vitamin D deficiency and two major oral health issues: tooth decay (dental caries [13,14,15]) and periodontitis [16]. Inadequate vitamin D levels can weaken teeth, making them more susceptible to cavities, fractures, and decay [17]. This susceptibility arises because insufficient vitamin D impairs the remineralization of the tooth structure, reducing the tooth’s ability to resist the acidic byproducts produced by cariogenic bacteria. Moreover, lower levels of vitamin D are associated with a higher risk of periodontitis, a severe gum disease that affects the tissues supporting the teeth. This increased risk is attributed to vitamin D’s role in modulating the immune system and its anti-inflammatory properties [18]. A deficiency can compromise the immune response, leading to increased inflammation and a greater likelihood of tissue breakdown and infection around the teeth [19].

In older adults, insufficient vitamin D impairs the absorption of calcium and phosphorus, adversely affecting the mineralization of tooth enamel and weakening the tooth’s natural defense mechanisms against cariogenic bacteria and acidic byproducts. In older adults, dentine consists of a narrower dentinal tubule that increases calcification, reduces the amount of peritubular fluid, and reduces sensitivity [20]. Additionally, older teeth have less water content, which leads to lower toughness and becoming more brittle [21]. While several studies demonstrate a potential association between vitamin D levels and the prevalence of dental caries [22,23], the evidence remains inconclusive. This ambiguity is likely due to variations in study design, differences in population selection, and inconsistencies in disease diagnosis criteria across different studies [24,25]. These methodological differences can lead to varied results, making it challenging to draw definitive conclusions about the relationship between vitamin D and dental caries.

Adding to the uncertainty is that most studies examining the association between vitamin D and dental caries have focused on children and younger adults. In contrast, there is a relative paucity of research investigating this relationship within the older adult population [26]. This disparity in focus is significant because findings from studies about children and younger adults may not apply to older adults due to differences in physiology, oral health conditions, and lifestyle factors that influence vitamin D levels and dental health. More targeted research is needed to clarify the potential connection between vitamin D levels and dental caries among older adults. Understanding this relationship is crucial as it may inform preventive strategies and interventions tailored to this demographic, which is particularly vulnerable to both vitamin D deficiency and dental caries. In light of this gap in the literature, this current study aimed to investigate the relation between the levels of vitamin D and the prevalence of dental caries in older adults. By doing so, it sought to provide a clearer understanding of whether there is an association between vitamin D levels and an increased risk of dental caries in this age group. The results could have significant implications for improving oral health outcomes and guiding nutritional and dental care recommendations for the aging population.

## 2. Methods

### 2.1. Data Source

This study analyzed raw data from the U.S. National Health and Nutrition Examination Survey (NHANES) spanning from 2011 to 2016 (accessible at https://wwwn.cdc.gov/nchs/nhanes/Default.aspx (accessed on 3 June 2024)). NHANES uses a multistage, cross-sectional, and complex survey design to represent the non-institutionalized civilian population across the United States. We focused on data from post-2011 because it includes detailed dental caries information and a race/ethnicity variable for Asian individuals, enhancing the precision of our analysis. The National Center for Health Statistics Research Ethics Review Board approved this study [27], and written consent was obtained from the parents of minor participants. More details about NHANES can be found on the Centers for Disease Control and Prevention website [28].

**Figure 1:**
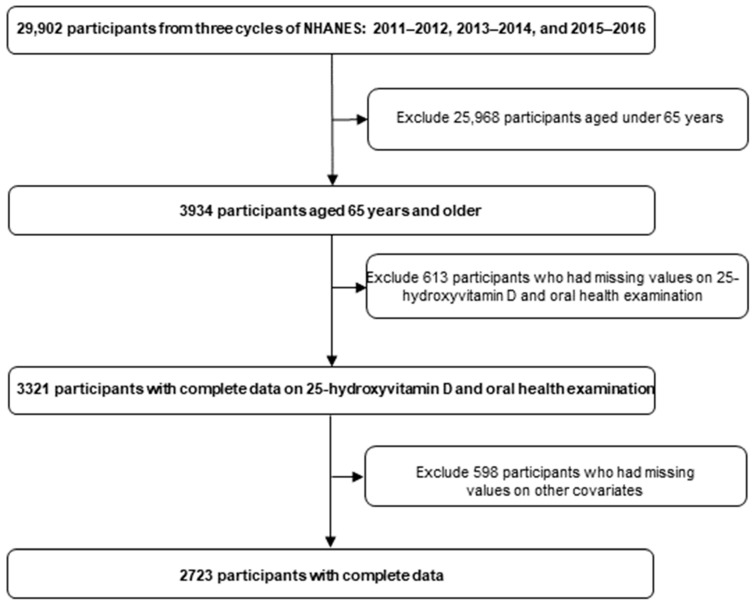
Sample selection criteria flowchart.

Our study included individuals aged 65 and older who completed both dental examinations and serum 25(OH)D tests, totaling 3321 individuals. After excluding 598 participants with incomplete data on other relevant variables, our final sample comprised 2723 individuals (Figure 1).

### 2.2. Measures

Licensed dentists, trained according to NHANES-specific protocols, conducted dental caries assessments using special equipment. The evaluation included two indicators: the decayed, missing, and filled teeth (DMFT) index and untreated dental caries. Untreated dental caries were identified by observing at least one tooth surface with a condition score ranging from 0 to 4 or any untreated carious root tips [29].

Vitamin D levels were measured as continuous variables representing serum 25(OH)D concentrations, which included combined levels of 25(OH)D2 and 25(OH)D3, measured using ultra-high-performance liquid chromatography–tandem mass spectrometry [30]. Serum 25(OH)D levels were classified into four categories: severely deficient (<25 nmol/L), deficient (25–49.9 nmol/L), insufficient (50–74.9 nmol/L), and normal (≥75 nmol/L), following established guidelines.

We included several demographic variables as covariates: age, gender, country of birth, and race/ethnicity. Gender was categorized as male or female. Race/ethnicity groups included non-Hispanic white, non-Hispanic Black, non-Hispanic Asian, other Hispanic, Mexican American, and other races. The country of birth was identified as either the United States or other countries.

### 2.3. Statistical Analyses

Continuous variables were summarized as mean ± standard deviation (SD), while categorical variables were presented as frequencies (percentages). Differences between categorical variables were evaluated using the Chi-square test. For continuous variables, the Mann–Whitney U test was employed to compare the two groups, and the Kruskal–Wallis test was employed for comparisons across more than two groups. Spearman’s rank correlation coefficient was used to evaluate the relationship between two continuous variables.

To investigate the association between vitamin D levels and untreated dental caries, we employed logistic regression models to provide odds ratios (ORs) with 95% confidence intervals (CIs). Poisson regression was employed to investigate the relationship between vitamin D levels and DMFT scores, with the results expressed as rate ratios (RRs). The analysis began with Model I, which assessed the unadjusted association between vitamin D levels and untreated dental caries. Model II expanded the analysis by adjusting for age, gender, race/ethnicity, and country of birth. These sequential models were used to evaluate the relationship between vitamin D levels and the presence of dental caries. Statistical significance was determined by a two-sided *p*-value of less than 0.05.

## 3. Results

This study analyzed data from 2723 participants across the NHANES cycles of 2011–2012, 2013–2014, and 2015–2016. The mean DMFT among older adults in the United States was 17.73 ± 8.34, with a prevalence of 35.1% for untreated dental caries. The participants had a mean age of 73.07 ± 5.35 years, with an equal distribution of females and males (50% each). Table 1 presents the bivariable analysis of untreated dental caries, showing significant associations with various demographic factors. Higher occurrences of untreated dental caries were found in males (38.1%) and Mexican American participants (48.7%). Additionally, higher rates were observed in participants with deficient vitamin D levels (40.9%) and those born outside the US (35.6%). Table 1 also presents the DMFT scores, showing significant variations across race/ethnicity and country of birth. Notably, multi-racial participants exhibited higher DMFT scores (19.87 ± 8.73). Higher scores were also observed in participants with severely deficient vitamin D levels (19.33 ± 8.56) and US-born individuals (18.06 ± 8.22). Spearman’s rank correlation coefficient revealed significant associations, indicating that increasing age correlated with higher DMFT scores.

Table 2 shows the distribution of serum 25(OH)D concentrations among Americans aged 65 years or older. Deficiency levels varied as follows: <25 nmol/L at 1.8%, 25–49.9 nmol/L at 15.1%, 50–74.9 nmol/L at 30.7%, and ≥75 nmol/L at 52.4%. Females more frequently had serum 25(OH)D concentrations below 25 nmol/L (2.4% versus 1.2% in males), but less frequently in the 25–49.9 nmol/L and 50-74.9 nmol/L ranges. Non-Hispanic whites had the lowest frequencies of 25–50 nmol/L (9.5%, *p* < 0.001), while Non-Hispanic Blacks had the highest frequencies below 25 nmol/L (4.8%) and 25-50 nmol/L (25.1%, *p* < 0.001). US-born individuals had higher frequencies of <25 nmol/L (2% compared to 1.1% in those born outside the US), but lower in the 25–49.9 nmol/L and 50-74.9 nmol/L categories. Finally, individuals with untreated caries showed the highest frequencies in <25 nmol/L, 25-49.9 nmol/L, and 50–74.9 nmol/L (2.1%, 17.6%, and 31.9%, respectively; *p* < 0.01).

Table 3 examines the relationship between vitamin D levels and dental caries. Individuals with vitamin D deficiency had a 1.44 times higher likelihood of having untreated caries (95% CI: 1.15, 1.81) compared to those with sufficient vitamin D levels. However, this association diminished after adjusting for demographic factors, resulting in an adjusted OR of 1.23 (95% CI: 0.97, 1.55). Additionally, severe vitamin D deficiency was associated with a 1.13 times higher rate of worse DMFT scores (95% CI: 1.06, 1.20), and this relation remained similar after adjustment, with an adjusted RR of 1.12 (95% CI: 1.05, 1.20).

## 4. Discussion

Most research examining vitamin D and dental caries focuses on children [31]. This study demonstrated a significant inverse relationship between vitamin D levels and DMFT among older adults and adds to the literature supporting a link between vitamin D and caries [31]. Specifically, vitamin D deficiency was associated with a higher likelihood of poorer DMFT scores.

The association between vitamin D deficiency and untreated dental caries highlights the complexity of vitamin D’s role in dental caries development. Initially, a link between vitamin D and untreated caries was observed. However, after adjusting for demographic variables, the association nearly reached statistical significance (adjusted OR of 1.23; 95% CI: 0.97, 1.55). This weakening of effect suggests that demographic factors such as age, gender, race/ethnicity, and country of birth influence the relationship between vitamin D and dental caries. This finding aligns with other research across various populations, emphasizing the multifaceted nature of dental caries, influenced by nutritional, genetic, and environmental factors [17].

In contrast, the relationship between severe vitamin D deficiency and higher DMFT scores remained significant after adjusting for confounding demographic variables. This indicates that vitamin D’s impact on dental health extends beyond its role in caries to include overall tooth health, encompassing missing and filled teeth. Vitamin D is crucial for calcium and phosphate metabolism and is essential for maintaining tooth structure and resistance to decay [12]. However, the precise mechanisms by which vitamin D affects oral health remain uncertain. In addition to its role in calcium metabolism and tooth mineralization, vitamin D also exhibits immunomodulatory effects [32] and antimicrobial activity, which may reduce inflammation and inhibit the growth of cariogenic bacteria [33]. A deficiency in older adults may additionally serve as a marker of long-standing deficiency, with higher DMFT scores reflecting accumulated damage over many years.

The high prevalence of vitamin D deficiency among the older adult population is concerning, especially given the increasing number of older adults and the associated health challenges of this demographic. Females had a higher frequency of severe vitamin D deficiency (<25 nmol/L) than males, aligning with the existing literature, suggesting that older women are at greater risk of vitamin D deficiency likely related to factors such as reduced dietary intake and less sun exposure [34]. This discrepancy emphasizes the need for targeted interventions to address vitamin D deficiency, particularly in older women.

Significant differences in vitamin D levels across race/ethnicity and country of birth further highlight the need for targeted public health interventions. Non-Hispanic Blacks had the highest frequencies of severe deficiency, which may be due to higher melanin levels reducing skin synthesis of vitamin D [35]. These disparities underscore the necessity for culturally sensitive and accessible nutritional guidance and supplementation programs to meet the specific needs of diverse populations.

The association between increasing age and higher DMFT scores underscores the cumulative effect of age on dental health. As individuals age, the likelihood of developing chronic conditions that affect oral health, such as reduced salivary flow, increased medication use, and general wear and tear on teeth, increases [36,37]. Therefore, maintaining adequate vitamin D levels in older adults could be a crucial component of comprehensive dental care strategies directed at improving the oral health of this population.

### 4.1. Strengths and Limitations

The strengths of this study include the use of a large, nationally representative sample from NHANES and a robust statistical analysis to minimize demographic confounders. However, the cross-sectional design precludes establishing causality between vitamin D levels and dental caries. Additionally, while the study controlled for several demographic factors, other potential confounders such as dietary habits, fluoride intake, toothbrushing, and socioeconomic status were not accounted for in the analysis. This study also does not provide insight into how early-life factors and vitamin D levels before age 65 might influence oral health in later years. Future research may consider these factors and employ longitudinal designs to understand the causal pathways better.

### 4.2. Public Health Implications and Future Research

This study examined Vitamin D and caries because of their role in calcium and phosphorus metabolism, minerals crucial for healthy teeth. Our findings suggest that maintaining sufficient vitamin D levels may play a role in preserving overall dental health among older adults. Others have identified vitamin D’s potential for preventing caries [25], and our findings suggest public health strategies that include vitamin D screening and supplementation may offer an opportunity to enhance oral health outcomes among high-risk older adults. The burden of dental disease among older adults in combination with vitamin D’s safety and low cost makes this a potentially high-yield strategy. Further research is needed to elucidate the mechanisms by which vitamin D influences dental health and to develop targeted interventions and specific recommendations to reduce the burden of dental caries in this vulnerable population.

## 5. Conclusions

In conclusion, the association between vitamin D deficiency and higher DMFT scores suggests a potential oral health benefit of effective public health strategies to address vitamin D deficiency. Ensuring sufficient vitamin D levels through dietary intake, supplementation, and adequate sun exposure could be a key factor for improving dental health and quality of life in older adults.

## Figures and Tables

**Table 1:** Prevalence of untreated caries and mean DMFT score by participant characteristics in the NHANES cycles 2011–2016.

Variables	All Participants	Participants with Untreated Caries Mean ± SD N (%)	Untreated Caries *p*-Value	Participants DMFT Score Mean ± SD	DMFT *p*-Value
Age (years)	73.07 ± 5.35	72.54 ± 5.31	--	73.16 ± 5.35	<0.001 ^b^
Sex					
Female	1362 (50%)	437 (32.10%)	0.008 ^a^	17.63 ± 8.27	0.58 ^c^
Male	1361 (50%)	519 (38.10%)		17.82 ± 8.40	
Race and Ethnicity					
Mexican American	265 (9.70%)	129 (48.70%)	<0.001 ^a^	15.17 ± 8.45	<0.001 ^d^
Other Hispanic	278 (10.20%)	104 (37.40%)		18.03 ± 8.16	
Non-Hispanic white	1434 (52.70%)	450 (31.40%)		17.93 ± 8.24	
Non-Hispanic Black	502 (18.40%)	194 (38.60%)		19.11 ± 7.88	
Non-Hispanic Asian	189 (6.90%)	59 (31.20%)		14.98 ± 8.90	
Other Race *	55 (2%)	20 (36.4%)		19.87 ± 8.73	
Birth country					
United States	2088 (76.70%)	730 (35%)	<0.001 ^a^	18.06 ± 8.22	<0.001 ^c^
Others	635 (23.30%)	226 (35.60%)		16.64 ± 8.64	
25(OH)D (nmol/mL)					
Normal (≥75)	1428 (52.40%)	463 (32.40%)	<0.001 ^a^	17.41 ± 8.24	0.002 ^d^
Insufficient (50–74.9)	835 (30.70%)	305 (36.50%)		17.58 ± 8.45	
Deficient (25–49.9)	411 (15.10%)	168 (40.90%)		18.96 ± 8.30	
Severely deficient (<25)	49 (1.8%)	20 (40.80%)		19.33 ± 8.56	

Note: * Includes multi-racial participants. ^a^ Chi-square test. ^b^ Spearman Rank Correlation. ^c^ Mann–Whitney U test. ^d^ Kruskal–Wallis test.

**Table 2:** Serum 25(OH)D concentrations in participants aged 65 years and older (N = 2723).

Variables	Severely Deficient (<25 nmol/L)	Deficient (25–49.9 nmol/L)	Insufficient (50–74.9 nmol/L)	Normal (≥75 nmol/L)	*p*-Value
Untreated caries					
Yes	20 (2.10%)	168 (17.60%)	305 (31.90%)	463 (48.40%)	0.008 ^a^
No	29 (1.60%)	243 (13.80%)	530 (30%)	965 (54.60%)	
DMFT Scores	18.94 ± 8.91	18.96 ± 8.30	17.58 ± 8.45	17.41 ± 8.24	0.002 ^b^

Note: ^a^ Chi-square test, ^b^ Kruskal–Wallis test.

**Table 3:** Association of vitamin D with untreated caries and DMFT scores in older adults.

Variables	Model I ^1^	Model II ^2^
Untreated Caries	OR [95% CI]	OR [95% CI]
Normal (≥75 nmol/L)	Reference	Reference
Insufficient (50–74.9 nmol/L)	1.20 [1.00–1.44] *	1.07 [0.89–1.29]
Deficient (25–49.9 nmol/L)	1.44 [1.15–1.81] *	1.23 [0.97–1.55]
Severely deficient (<25 nmol/L)	1.44 [0.81–2.57]	1.20 [0.66–2.18]
DMFT Scores	RR [95% CI]	RR [95% CI]
Normal (≥75 nmol/L)	Reference	Reference
Insufficient (50–74.9 nmol/L)	1.03 [1.00–1.05] *	1.04 [1.02–1.06] *
Deficient (25–49.9 nmol/L)	1.08 [1.05–1.11] *	1.09 [1.07–1.12] *
Severely deficient (<25 nmol/L)	1.13 [1.06–1.20] *	1.12 [1.05–1.20] *

Note. ^1^ Model I was unadjusted. ^2^ Model II adjusted for age, gender, race/ethnicity, and country of birth. * *p* < 0.05.

## Data Availability

The data presented in this study are openly available at https://wwwn.cdc.gov/nchs/nhanes/Default.aspx (accessed on 3 June 2024).
